# Biallelic and Genome Wide Association Mapping of Germanium Tolerant Loci in Rice (*Oryza sativa* L.)

**DOI:** 10.1371/journal.pone.0137577

**Published:** 2015-09-10

**Authors:** Partha Talukdar, Alex Douglas, Adam H. Price, Gareth J. Norton

**Affiliations:** Institute of Biological and Environmental Sciences, University of Aberdeen, Aberdeen, AB24 3UU, United Kingdom; Università Politecnica delle Marche, ITALY

## Abstract

Rice plants accumulate high concentrations of silicon. Silicon has been shown to be involved in plant growth, high yield, and mitigating biotic and abiotic stresses. However, it has been demonstrated that inorganic arsenic is taken up by rice through silicon transporters under anaerobic conditions, thus the ability to efficiently take up silicon may be considered either a positive or a negative trait in rice. Germanium is an analogue of silicon that produces brown lesions in shoots and leaves, and germanium toxicity has been used to identify mutants in silicon and arsenic transport. In this study, two different genetic mapping methods were performed to determine the loci involved in germanium sensitivity in rice. Genetic mapping in the biparental cross of Bala × Azucena (an F_6_ population) and a genome wide association (GWA) study with 350 accessions from the Rice Diversity Panel 1 were conducted using 15 μM of germanic acid. This identified a number of germanium sensitive loci: some co-localised with previously identified quantitative trait loci (QTL) for tissue silicon or arsenic concentration, none co-localised with *Lsi1* or *Lsi6*, while one single nucleotide polymorphism (SNP) was detected within 200 kb of *Lsi2* (these are genes known to transport silicon, whose identity was discovered using germanium toxicity). However, examining candidate genes that are within the genomic region of the loci detected above reveals genes homologous to both *Lsi1* and *Lsi2*, as well as a number of other candidate genes, which are discussed.

## Introduction

Silicon is an important nutrient for rice, which enhances yield [1,2] and alleviates biotic and abiotic stresses [3]. Three silicon transporters (*Lsi1*, *Lsi2*, and *Lsi6*) which are involved in the uptake, distribution, and accumulation of silicon in rice have been identified [3–5]. *Lsi1* and *Lsi2* genes control the transport and distribution of silicon in rice, and are located on chromosome 2 and 3 respectively. *Lsi1* (OsNIP2;1) is categorised as an influx transporter, whereas *Lsi2* is categorised as an efflux transporter of silicon in rice [3,4]. In addition, *Lsi6* (OsNIP2;2) is localised on chromosome 6 and regulates silicon distribution in rice shoots [5]. In a recent study, using a modelling approach, the importance of the casparian strip in silicon uptake in rice was identified (6). In addition, in that study it is speculated that there could be more transporters involved in silicon transport, for example unknown transporters on the pericycle cells [6]. Inorganic arsenic, which is a class one carcinogen, is found in rice grains in two species: arsenate and arsenite. Arsenate is an analogue of phosphate [7] while arsenite is taken up by silicic acid transporters in rice (8). It has been demonstrated that arsenate is reduced to arsenite within the rice root [9,10]. Arsenite can then enter the xylem via a silicic acid / arsenite effluxer, *Lsi2* [8,10]. Germanium is a toxic element to plants, and symptoms include lesions of brown spots on leaves [11,12]. Germanium is an analogue of silicon, since plant roots uptake germanium as Ge(OH)_4_ using the same mechanism as silicon, which is taken up in the form of Si(OH)_4_ [13,14]. Takahashi *et al*. (1976a & 1976b) explained that the uptake kinetics of germanium in plant roots is similar to silicon [11,12]. The subcellular localisation of silicon and germanium in root and shoot tissue of blue grass (*Poa annua* L.) and orchard grass (*Dactylis glomerata* L.) has been determined [15]. In root tissue silicon and germanium were present in the suberised, thick walled region of endodermal cells. In the leaves germanium was barely detectable; however silicon was located in the cell walls. The presence of silicon and germanium in the proximal side of the endodermal cell walls of roots indicates that there is a control of silicon and germanium uptake by the plant [15]. In the leaves of the plants, the low concentrations of germanium but higher concentrations of silicon indicate that there is preferential transport of silicon relative to germanium [15].

A number of studies have been conducted previously where germanium was used to trace silicon, boron, or arsenic in rice and other monocots. For example, the lack of appearance of germanium-induced lesions was used to identify the *Lsi1* and *Lsi2* mutants in rice [3,4]. In addition, germanium was used to examine silicic acid and arsenite competition in shoot-to-grain transport in rice species [16]. Germanium has also been used to conduct studies exploring natural variation. Two quantitative trait loci (QTL) have been detected in barley using germanium toxicity; one of the QTLs co-localised with HvNIP2;1 an aquaporin with permeability to boron, silicon, arsenic, and germanium. This indicates the possible use of germanium to trace candidate genes in the genome [17].

Since germanium is an analogue of both silicon and arsenite, studies on the genetics of germanium toxicity may provide insights into the genetics of silicon and arsenite uptake. Previously a number of studies have conducted genetic mapping to identify silicon and arsenic QTLs in rice. A study by Norton *et al*., (2010) identified five QTLs for shoot arsenic concentration and two QTLS for shoot silicon concentration in the F_6_ Bala x Azucena mapping population grown in Wuhan, China [18]. The same population was also grown in Qiyang, China, where a number of QTLs for grain arsenic concentration were identified [19]. In a study by Zhang *et al*., (2008) using the CJ06 x TN1 population, four arsenic QTLs were identified (one for shoot, one for root, and two for grain arsenic concentration) [20]. A total of five QTLs for grain arsenic concentration were identified in a backcross introgression population (TeQing x Lemont), with one of these QTLs also identified in a recombinant inbred line (RIL) population generated from the same parents [21]. Genome wide association (GWA) mapping has also been conducted for grain arsenic using the Rice Diversity Panel 1 (RDP1), at four different field locations (two in the USA, one in Bangladesh, and one in China) [22]. From that study a large number of loci associated with grain arsenic concentration were identified. As well as the identification of QTLs for total arsenic concentration, QTLs for the rice grain concentration of one of the organic arsenic species, dimethylarsinic acid, have been identified [23]. It has been demonstrated that the organic arsenic species in plants come from the environment rather than being biosynthesised *in planta* [24,25]. Evidence suggests that microorganisms in the soil mediate the methylation of arsenic [24,25]. Therefore these QTLs could be linked to rhizosphere methylation [23]. A total of three QTLs for rice grain dimethylarsinic acid concentration were identified [23]. It has been demonstrated that *Lsi1* mediates the influx of monomethylarsenate (MMA(V)), and to a lesser extent, dimethylarsenate (DMA(V)), into rice roots [26]. QTLs for silicon concentration in different parts of rice plants have been identified from a cross of Zhenshan 97B x Milyang 46 [27]. A total of four QTLs were identified for hull silicon concentration, four for flag leaf silicon concentration, and two for stem silicon concentration. A number of these QTLs co-localised. On chromosome 1 near marker RM151 a hull silicon QTL co-localises with a stem silicon QTL. Also on chromosome 1 near marker RM246, a hull silicon QTL co-localises with a flag leaf silicon QTL. On chromosome 11 a hull silicon QTL co-localises with a flag leaf silicon QTL [27]. Furthermore, Wu *et al*., (2006) mapped QTLs for silicon uptake and silicon uptake per unit of root dry weight, revealing three and four QTLs for the respective traits [28]. A total of six silicon concentration QTLs were identified in rice hulls using association mapping of a mini-core collection of 174 accessions, genotyped with 164 markers [29]. Information on the natural variation of silicon transporters in rice may help in strategies to reduce the grain arsenic accumulation in rice.

In this study, a germanium induced lesion phenotype was used to advance understanding of genetic variation in arsenic and silicon uptake in rice. Initial experiments were conducted to develop a quantitative high throughput phenotyping method. This was then used to reveal loci in a bi-parental F_6_ (Bala × Azucena) mapping population and the RDP1. QTLs and significant SNPs were compared with previous studies on silicon and arsenic accumulation. From these studies a number of candidate genes were identified. Finally, an experiment was conducted to compare germanium induced lesions with tissue concentrations of germanium.

## Materials and Methods

### Genotypic difference of germanium accumulation and lesions in two rice cultivars

In the first experiment, Azucena (*tropical japonica*) and Bala (*indica*) seeds were surface sterilised in 1% sodium hypoclorite and kept at 30°C for three days to allow for germination. Half strength nutrient solutions, pH 5.5 [30] containing 0, 1.5, 5, 15, 50, 150, and 500 μM germanium acid were prepared, by dissolving germanium dioxide in water. Five germinated seeds were placed on a net (2 × 1.5 cm) which was floated on polyethylene beads in plastic cups (250 mL) containing the various germanium solutions. Germanium causes the development of brown lesions on the rice shoots and leaves. The visible plant symptoms for germanium induced lesions were recorded after one week based on the percentage of the leaves and shoots affected by brown lesions and necrosis (S1 Fig).

In a second experiment, Azucena and Bala seeds were germinated as previously described. Two 20 L plastic boxes (34 × 27 × 22 cm) were filled with half strength nutrient solutions, pH 5.5 [30]. A plastic multi-cell tray consisting of 154 wells (11 row × 14 columns) was placed in each box, and ten replicates of each genotype (10 Azucena and 10 Bala) were randomly placed in each tray. After one week the nutrient solution was changed; in one box it was kept as half strength nutrient solution while in the other box it was half strength nutrient solution with 15 μM germanium acid. The lesions were quantified as previously described, from the second day after the solution was changed until the seventh day.

### Mapping germanium tolerant loci in Bala × Azucena F_6_ population

The rice mapping population used in this study was an F_6_ RIL population derived from a cross between two cultivars, Bala and Azucena [31]. The experiment was conducted on 132 of the RILs. Three plastic boxes (28 L capacity: 52 × 33 × 15.8 cm) were used in this experiment, with two plastic multi-cell trays consisting of 154 wells (11 row × 14 columns) in each box to hold the seeds. Every RIL was randomised in each box (three replicates) with three seeds in each well. After the seeds had germinated the rice seedlings were thinned to one seedling per well. A plastic mesh was used in each well to hold seeds on the tray, and the water level was kept up to a height were it just touched the seeds. Initially the seeds were germinated and the seedlings grown in half strength Yoshida’s solution for 14 days with the nutrient solution changed after 7 days. After 14 days the nutrient solution was changed to a nutrient solution containing 15 μM germanic acid. The solution was then changed every day with fresh nutrient solution containing 15 μM germanic acid until the end of the experiment. Germanium lesion scoring started on the 4^th^ day after germanium addition and continued for the next four days. Traits were named based on the day that they were quantified: Ge4 quantified on day 4, Ge5 quantified on day 5, Ge6 quantified on day 6, Ge7 quantified on day 7, and Ge8 quantified on day 8.

The QTL analysis was carried out as described in Price *et al*., (2002) [32]. The molecular map of Azucena and Bala RIL populations contains 164 markers covering 1,833 cM on 12 linkage groups [33]. The QTL identification was conducted by composite interval mapping using QTL Cartographer version 1.15 [34]. Permutation analysis implemented in QTL Cartographer 1.15, with 1000 replications, was used to determine the genome wide 5% and 10% significance levels for QTL detection using the log odds (LOD) criterion. In this study, a 5% genome wide threshold was used to identify QTLs and a 10% threshold was used to identify putative QTLs. The broad sense heritability (H^2^) was calculated using the *F* value from the ANOVA.

To determine if there were any epistatic interactions for the traits a mixed-model approach was used using the default settings of background genetic variation and interaction markers of the programme QTLMapper version 1 [35,36]. To further confirm the interactions a two-way analysis of variance using the marker genotypes was performed.

### GWA mapping of germanium tolerant loci in the Rice Diversity Panel 1

The population used for the genome wide association mapping was the RDP1 [37–39]. Within the RDP1 there are cultivars which represent the five subpopulations of rice (*indica*, *aus*, *tropical japonica*, *temperate japonica*, *and aromatic (group V)*). The cultivars were genotyped using an Affymetrix SNP array containing 44,100 SNPs, with ~1 informative SNP per 10 kb [40]. In this study, 341 cultivars of *O*. *sativa* from RDP1 were screened for germanium tolerance.

The experiment was conducted in the same way as the Bala × Azucena F_6_ mapping population experimental set up, except for the approach to replication. Every cultivar was randomised into one of three different boxes (28 L) and two seeds were used in each well; these were thinned to one plant per well once the seedlings had germinated. Experiments were conducted in four replicate runs at different times, with all genotypes present in each replicate run. After the plants had been exposed to germanic acid, as described above, they were phenotyped as before based on the percentage of the shoot covered in lesions.

GWA mapping was performed using a mixed model approach on all the cultivars implemented, using EMMA (Efficient Mixed Model Analysis) [41], and was also performed within four different subpopulations (*aus*, *indica*, *temperate japonica and tropical japonica*) as described in Norton *et*. *al*.,(2014) [22].

A SNP was selected as significantly associated with the trait if the *P-*value was < 0.0001 and if the minor allele frequency (MAF) was > 5% [22]. Significant SNPs within 200 kb of each other were considered to represent SNP clusters of the same QTL. Gene annotation was examined 200 kb either side of a significant SNP or SNP cluster.

### Genotypic difference of germanium uptake and lesions in rice cultivars

An experiment was conducted to test the hypothesis that the genetic variation of germanium lesions in rice cultivars is associated with germanium accumulation. The cultivars for this experiment were selected on the basis of germanium sensitivity from the association mapping experiment. A total of 8 cultivars from the *aus* subpopulation (4 from high and 4 from low germanium sensitive) and 8 from the *temperate japonica* subpopulation (4 from high and 4 from low germanium sensitive) were chosen. A cultivar (DJ 123 (*aus*)) was also selected which had a medium germanium toxicity lesion in GWA analysis. In addition, Azucena and Bala were also included in the experiment. Four replicates of each rice cultivar were set up in a 28 L box as previously described, containing two seeds from each cultivar in each well; these were thinned to one seedling after germination. All the empty wells were filled with either Azucena or Bala seeds. Plants were grown and treated with 15 μM germanium as described previously. Every plant was quantified for germanium lesions after 4 days and then collected for germanium analysis. Samples were dried in oven at 75°C for 3 days.

A total of approximately 0.02 g of dried sample was accurately weighed into 50 mL polyethylene centrifuge tubes, and 0.4 mL concentrated (70%) nitric acid was added to the sample and left overnight at room temperature. After overnight incubation 0.4 mL hydrogen peroxide (30%) was added immediately prior to microwave digestion [42]. Total germanium concentrations for digested samples were determined by Inductively Coupled Plasma Mass Spectrometry (ICP-MS) (Agilent Technologies 7500) [42]. Trace-element grade reagents were used for all digests, and for quality control replicates of certified reference material (CRM) and blank digestions were used. Rhodium (10 μg L^-1^) was run on an external line as the internal standard.

### Identification of candidate genes

Two approaches were used to identify candidate genes from the genetic mapping experiment. The first approach was identifying the genes within either 5 cM of the QTL peak for the Bala x Azucena genetic mapping experiment or within 200 Kb either side of the associated marker(s) for the association mapping. These distances were chosen based on previous studies [22,40,43]. Using the annotation of the genes within the target regions (taken from the rice genome annotation project (http://rice.plantbiology.msu.edu/)) functional position candidate genes were identified. The second approach identified homologues of known silicon uptake genes *Lsi1*, *Lsi2*, and *Lsi6* to identify if they were in the designated candidate gene regions. The protein sequences of these three genes were used to perform a BLAST search across all the rice genes.

### Statistics

Statistical analyses were conducted using Minitab 16 applying General Linear Models (GLM). QTL mapping and GWA mapping were conducted as described above.

## Results

### Genotypic difference of germanium induced lesions in Bala and Azucena

**Fig 1:**
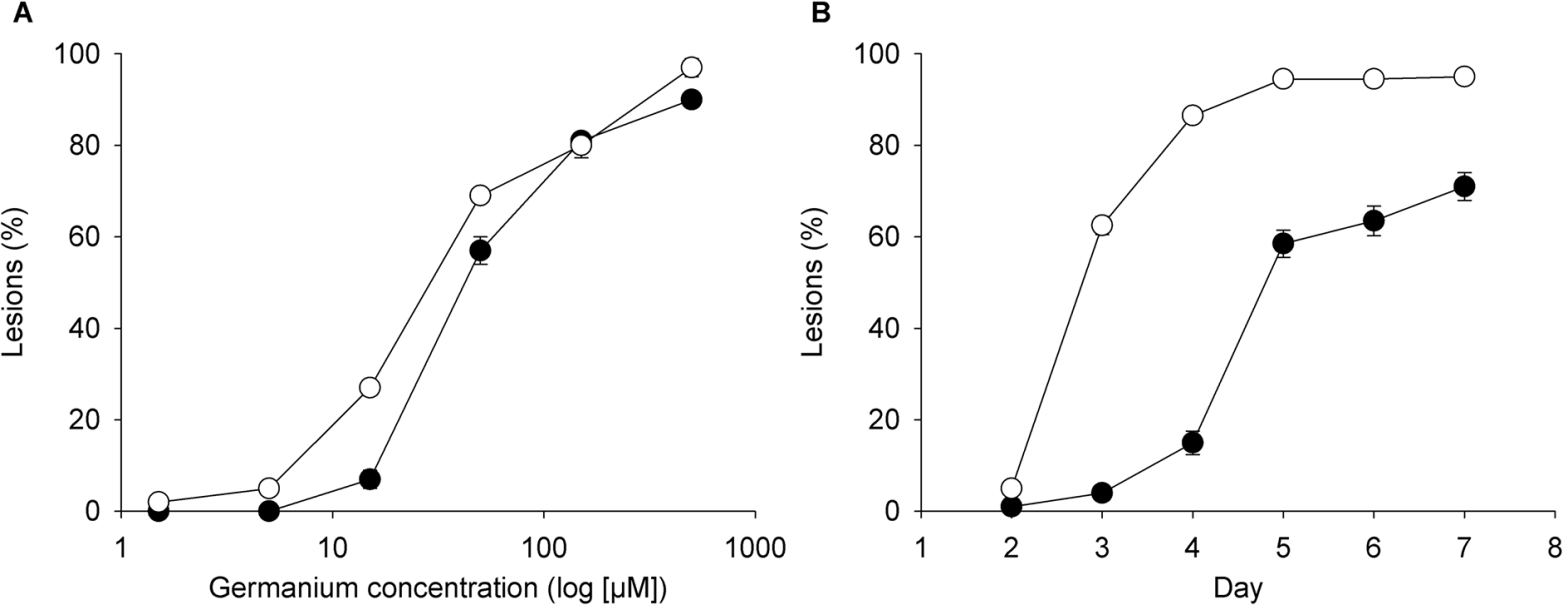
A) Lesions in Azucena (filled symbols) and Bala (open symbols) after one week in half strength nutrient solution containing different concentrations of germanium. B) Development of germanium lesions in Azucena (filled symbols) and Bala (open symbols) when exposed to 15 μM of germanium in half strength nutrient solution. Points represent the mean value for each treatment and the error bar represents the standard error of the mean.

Rice cultivars Azucena and Bala showed differences in the number of germanium induced lesions after 7 days in different germanium acid treatments (Fig 1A). Statistical analysis showed that there were highly significant differences of lesions between treatment levels (*P*<0.001) and between the two cultivars (*P*<0.001), and there was a cultivar by germanium treatment interaction (*P*<0.001). Germanium lesions were observed in Bala at 1.5 μM and increased as germanium concentration increased; the germanium lesions increased so that the mean percentage was 97% (almost all leaf and stem area covered with lesions) at 500 μM (Fig 1A). Azucena cultivars did not show any lesions when exposed to 1.5 and 5 μM germanic acid. It was also notable that when the concentration of germanic acid was 150 μM or above the difference between the percentage of lesions on Azucena and Bala decreased, and was found to be very similar between the cultivars. The rate at which the lesions developed was significantly different for the two cultivars as demonstrated for the 15 μM germanium acid treatment (Fig 1B). Statistical analysis of this treatment level shows that there was a significant genotypic difference (*P*<0.001) of germanium lesion between Azucena and Bala. Bala developed germanium lesions more rapidly than Azucena after the second day of germanium addition. The maximum difference between two genotypes in lesions at 15 μM germanic acid was observed on day 4, where Azucena and Bala had a mean of 15% and 86% lesions respectively; there was a clear difference of lesions between the two cultivars until day 7.

### Mapping germanium tolerant loci in Bala × Azucena F_6_ population

A total of 132 F_6_ RILs derived from Bala and Azucena were screened with 15 μM of germanium oxide in a hydroponic system. Bala had significantly (*P*<0.015) more lesions than Azucena on days 4, 5, 6, and 7. The maximum significant (*P*<0.001) difference in percentage coverage of lesions between Azucena and Bala was found on day 4, whereas there was no difference in percentage coverage of lesions on day 8 (*P*<0.087). The mean number of germanium induced lesions across the whole population increased each day, with the mean of 21.9% on day 4 and 60.1% on day 8 (Table 1, S2 Fig). The broad sense heritability for the trait was high, being highest (86.1%) for trait Ge4, whereas the lowest (75.8%) was for Ge8 (Table 1).

**Table 1:** Germanium induced lesions for the recombinant inbred lines on day 4, 5, 6, 7, and 8 after treatment with 15 μM germanic acid. There were significant differences (*P*< 0.001) between the recombinant inbred lines on each of the days.

Trait	Mean (%)	Minimum (%)	Median (%)	Maximum (%)	Heritability (H^2^) %
Ge4	21.9	0.0	20.0	55.0	86.1
Ge5	39.4	0.0	40.0	90.0	81.0
Ge6	52.7	5.0	50.0	90.0	82.9
Ge7	56.9	10.0	55.0	90.0	78.4
Ge8	60.1	15.0	60.0	100.0	75.8

**Fig 2:**
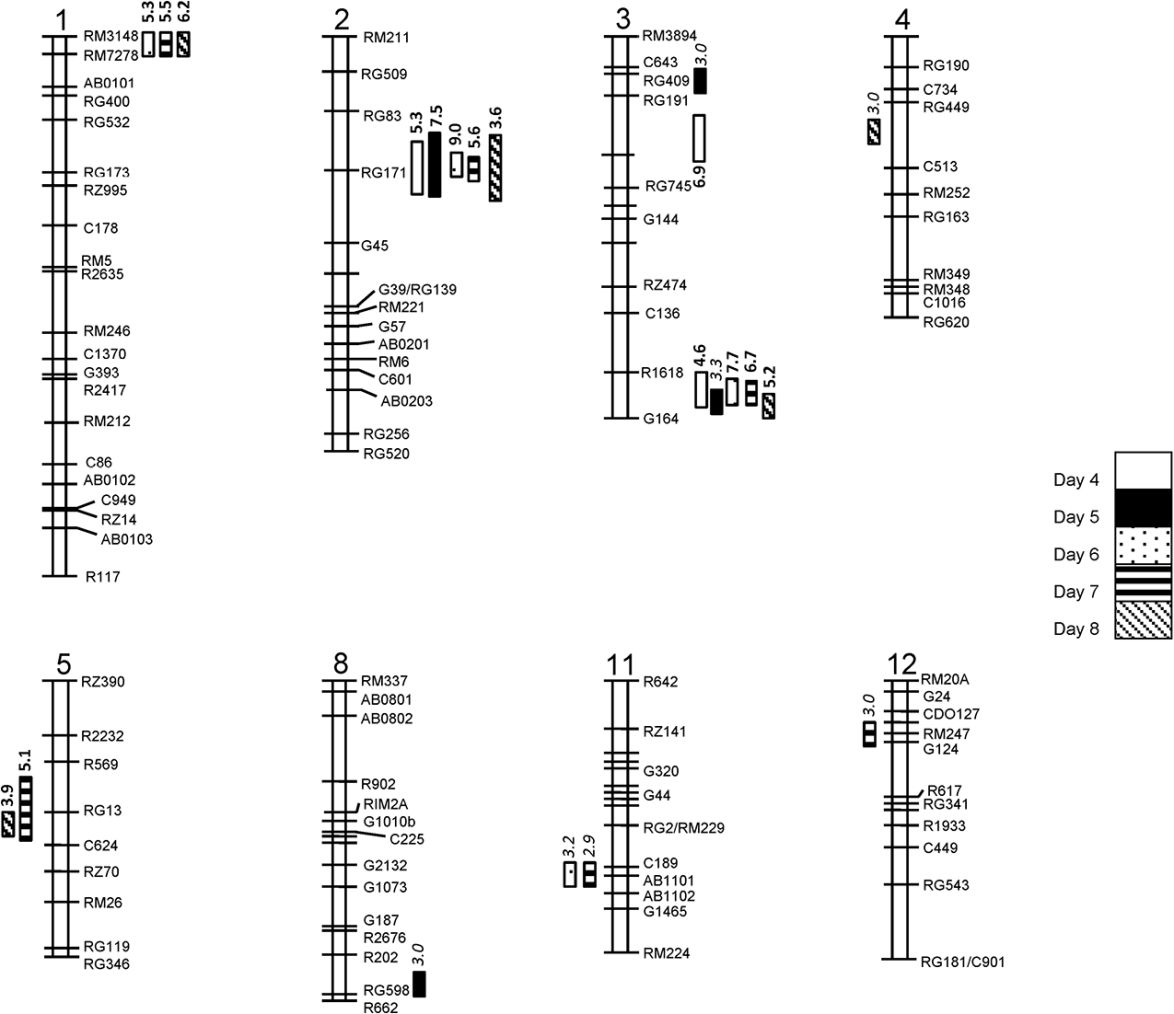
QTLs for germanium lesions in Bala × Azucena recombinant inbred lines on 8 of the 12 chromosomes. QTLs on the right-hand side of the chromosomes indicate the positive allele is from Bala (where the positive allele leads to an increase in lesions) whereas QTLs on the left-hand side of the chromosomes indicate the positive allele is from Azucena. Each QTL box denotes the 1 LOD range, with the value above or below the box denoting the LOD score. The LOD scores in bold indicate identification of QTLs, while LOD scores in italic indicate putative QTLs.

A summary of all significant and putative QTLs obtained from the data are presented in S1 Table. All QTLs are presented graphically on the linkage map in Fig 2. Putative QTLs with a LOD score of 10% threshold are also presented in Fig 2.

A QTL was detected on the top of chromosome 1, near marker RM7278, which accounted for 14, 15, and 18% of the trait variation on days 6, 7, and 8 respectively, with Bala being the donor of the positive allele (where the positive allele leads to an increase in lesions). A QTL detected on chromosome 2 around marker G45, also with a positive effect from the Bala allele, accounted for 13, 26, 19, 12, and 10% of the trait variation on days 4, 5, 6, 7, and 8 respectively.

Three QTLs (one of which is a putative QTL) were detected on chromosome 3. One was detected 24 cM above RG745, with a LOD score of 6.9, and explained 25% of the variation of the trait on day 4, where the positive effect came from the Bala allele. A QTL on chromosome 3 was near marker G164 with a positive effect from the Bala allele. The QTL was detected on every day and explained 14, 10, 17, 21, and 17% of the variation on days 4, 5, 6, 7, and 8 respectively, but it is noteworthy that this QTL was observed only as a putative QTL on day 5. In addition, there was another putative QTL detected on chromosome 3 on day 5, which explained 8% of the variation; the putative QTL was located near marker RG191 with the positive effect allele coming from Bala.

A putative QTL was detected on chromosome 4, with the Azucena allele having a positive effect on the trait. The QTL was mapped at approximately 50 cM and explained 13% of the variation. QTLs detected on chromosome 5, with the Azucena allele having a positive effect on the trait near to marker C624, accounted for 9% and 13% of variation, on day 7 and day 8 respectively. A putative QTL was mapped on chromosome 8 at 144 cM, contributed by the Bala allele, and explained 7% of the variation. A putative QTL was also detected on chromosome 11 on days 6 and 7 near marker AB1101, explaining 5% of the variation on both days, with the positive effect contributed by the Azucena allele. On chromosome 12, at 25 cM, a putative QTL was mapped, with Azucena contributing the positive allele; the QTL accounted for 6% of the observed variation.

Two significant epistatic interactions were detected for the trait on day 4. The first interaction was observed between markers RG532, chromosome 1 and AB0801, chromosome 8, with a LOD score of 3.8. The second observed interaction was between marker RG745 and marker R1618 both on chromosome 3, with a LOD score of 5.1. However, analysis of variance for both these interactions was not significant.

### GWA mapping of germanium tolerant loci in RDP1

Three hundred and forty one *O*. *sativa* accessions from the RDP1 were examined for germanium tolerance using the germanium toxicity test at 15 μM germanium oxide. The subpopulation breakdown for these accessions was; 12 *aromatic*, 55 *aus*, 62 *indica*, 79 *temperate japonica*, 82 *tropical* japonica, and 51 admix. One way ANOVA indicates that genotype explains 66.3%, 64.0%, and 60.3% of the variation for the germanium induced lesion trait on days 4, 5, and 6 respectively. For the trait measured on day 4 the germanium induced lesions ranged from 1.0%- 75.0%, on day 5 it ranged from 7.0%- 95.0%, and on day 6 it ranged from 22.0%- 97.0% (S3 Fig).

When the analysis of germanium induced lesions was done across the five subpopulations there were significant differences between the subpopulations on all three days (*P*<0.001). The amount of variation explained by this analysis was 18.0%, 17.1%, and 18.5% on days 4, 5, and 6 respectively. On days 4 and 5 the observed trend was that cultivars from the *aromatic* subpopulation had less germanium induced lesions than other subpopulations, followed by *temperate japonica* > *tropical japonica* > *aus* and *indica* (S4 Fig). However, on day 6 cultivars from the *aromatic* subpopulation had less germanium lesions, with cultivars from the *temperate japonica* subpopulation having the next lowest number of germanium induced lesions, and with the other three subpopulations all having similar numbers of germanium induced lesions.

**Fig 3:**
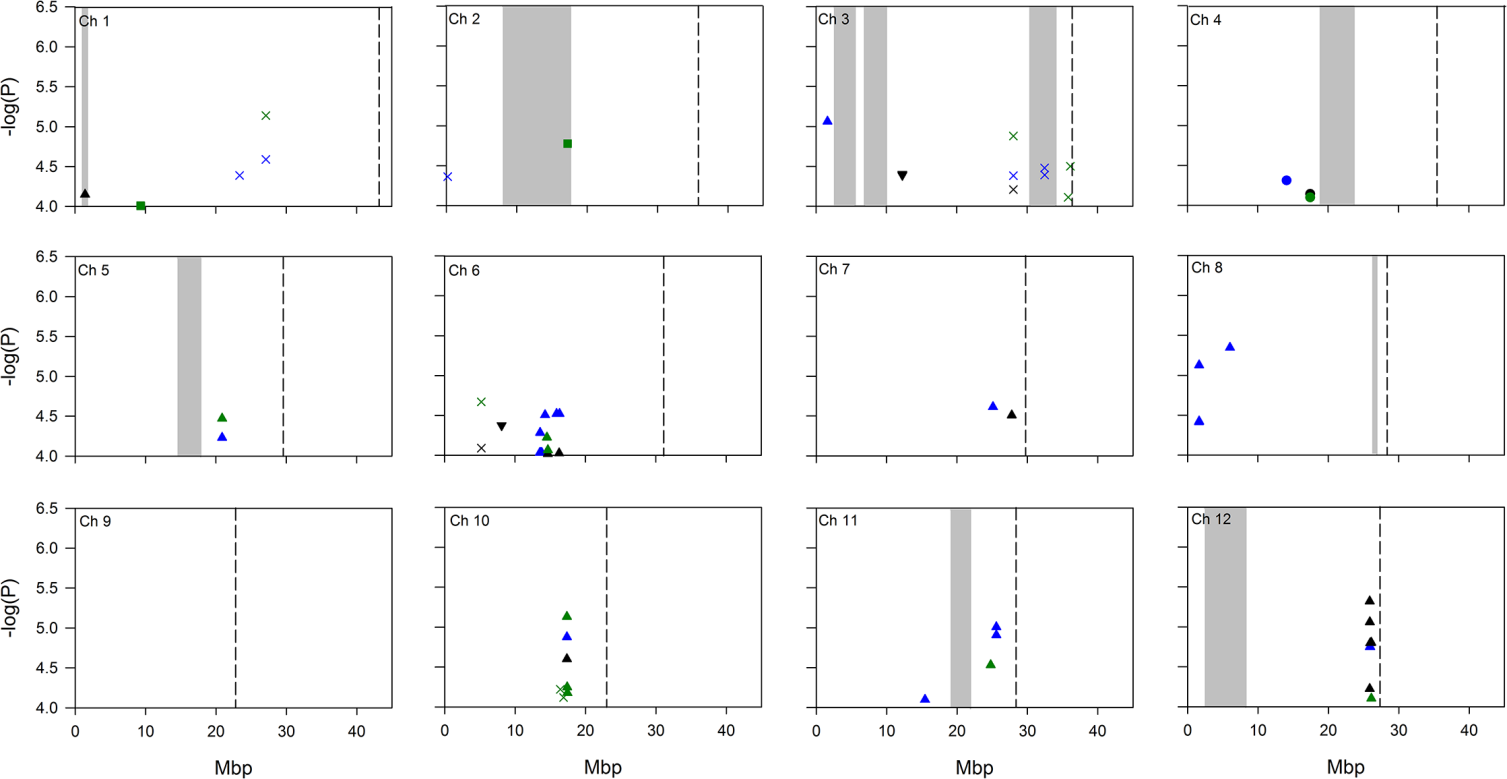
GWA mapping of germanium induced lesions across the 12 rice chromosomes. Note the Y axis starts at 4, so only the most highly associated SNPs are represented. Data points are SNPs significantly associated (*P*<0.0001) with the trait and which have a MAF > 5%. Significant SNPs from different days are displayed with different coloured symbols: day 4 are black, day 5 are green, and day 6 are blue. Analyses of the combined subpopulation groups and separate subpopulations are represented by different symbols: combined analysis = X, *aus* = circle, *indica* = square, *tropical japonica* = triangle, *temperate japonica* = inverted triangle. Grey highlighted bars indicate regions of mapped QTLs for germanium induced lesions identified in this study. Dotted lines indicate chromosome ends.

From the genome wide association mapping a total of 17 significant SNPs (*P*-value <0.0001, MAF > 0.05) were detected on days 4, 5, and 6 in the analysis using all accessions (the X symbols in Fig 3, see also S2 Table). A number of the SNPs significantly associated with the traits were observed on more than one day; significant SNPs id1015789 and id1015794 were detected at 27.10 Mb on chromosome 1 on both days 5 and 6. On chromosome 3 two loci were observed on multiple days. A SNP (id3012850) at 28.02 Mb was observed on days 4, 5, and 6, whereas the SNP id3015629 at 32.46 Mb on the same chromosome was observed on days 5 and 6. In addition, at 5.21 Mb on chromosome 6 significant SNP id6003502 was observed in the all analysis on days 4 and 5.

There were 54 SNPs detected when analysing subpopulations separately (S3 Table). The highest number (45) of significant SNPs was detected in *tropical japonica* analysis, whereas four SNPs were detected in *aus*, and three and two were detected in *temperate japonica* and *indica* respectively. There were multiple SNPs which were detected on more than one day in different subpopulations. On chromosome 4 a significant SNP (id4005078) was detected on both day 4 and day 5 at 17.43 Mb within the *aus* subpopulation. At 20.88 Mb on chromosome 5 a significant SNP (id5008807) was detected in the *tropical japonica* accessions on days 5 and 6. On chromosome 6 a significant SNP (id6008763) was detected on both days 4 and 5 at 14.66 Mb within the *tropical japonica* subpopulation. At 17.34 Mb on chromosome 10 a significant SNP (id10000497) was detected in the *tropical japonica* accessions son days 4, 5, and 6. On chromosome 12 three significant SNPs (id1200947, id1200950, and id1200965) were detected on more than one day within the *tropical japonica* subpopulation; these three SNPs are located between 25.89 and 26.10 Mb.

### Genotypic difference of germanium uptake and lesion in RDP1

**Fig 4:**
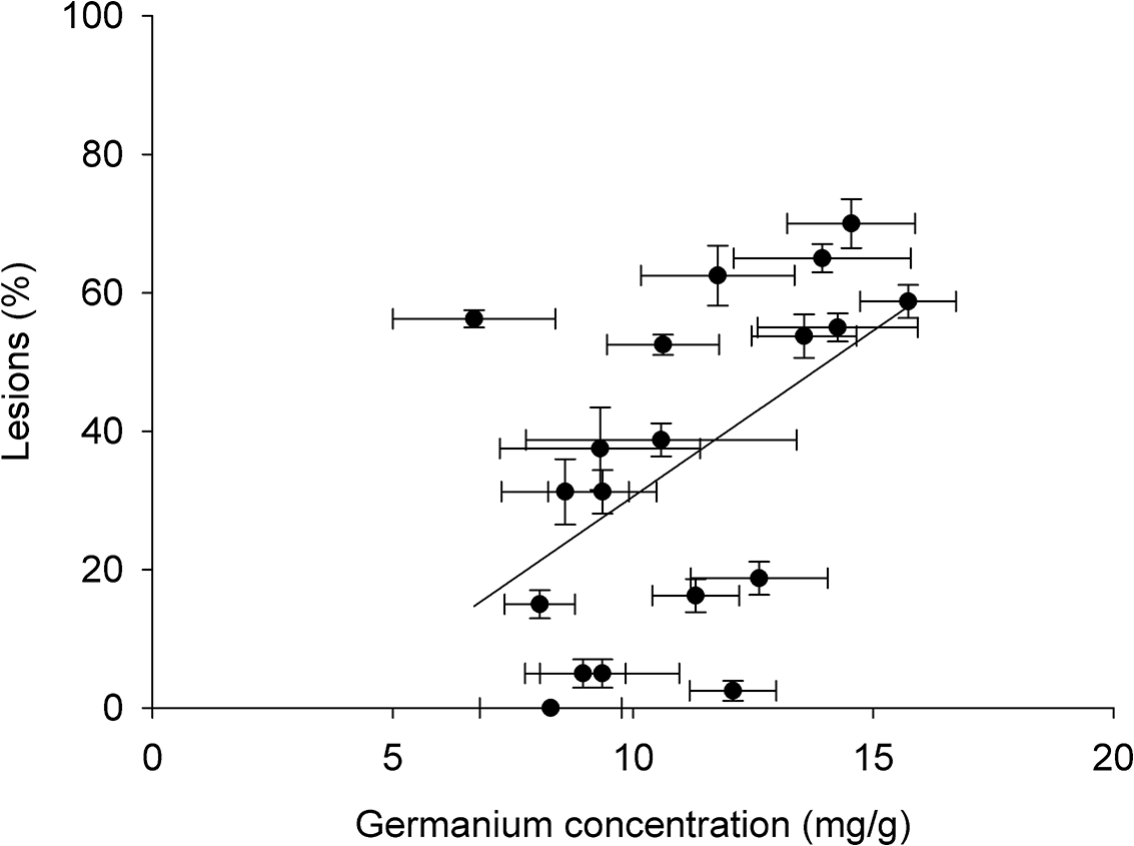
Plot of mean germanium accumulation (leaf germanium concentration) and mean germanium induced lesions for 19 cultivars after four days’ exposure to 15 μM germanium oxide solution. Each point represents the means value of a cultivar; error bars are standard error of the mean. Regression line is plotted (*r* = 0.477, *P* = 0.022).

An experiment was conducted to examine the relationship between genotypic differences in tissue germanium accumulation and germanium induced lesions in a set of 19 cultivars. Germanium accumulation ranged approximately 2 fold between the cultivars, and varied significantly between cultivars (*P*<0.001). A significant positive relationship was observed between germanium lesion and germanium accumulation (*R* ^*2*^ = 22.8%, *P* = 0.022, *F* = 6.33) (Fig 4).

### Candidate genes for germanium induced lesions

From all the identified positional candidate genes (S4 Table) a number of functional candidate genes were identified. These included genes annotated as transporters, with genes specifically annotated as ABC transporters and an aquaporin gene. Two of these candidate genes are particularly noteworthy as they have a high degree of homology with genes previously identified to be involved in silicon uptake and transport in rice. One of these genes (Os01g02190) is on chromosome 1 at 0.64 Mb; this gene has 60.1% homology with *Lsi1* and 94.1% homology with *Lsi6*. It is in the candidate gene list for a QTL detected in the Bala x Azucena mapping population and annotated as an aquaporin. Another gene (Os10g31040), on chromosome 10 at 16.23 Mb, annotated as a citrate transporter, is present in the candidate gene list for a QTL detected in the all analysis of the association population. This gene has 81.4% homology with *Lsi2*.

## Discussion

A rapid quantitative method for the assessment of germanium induced lesions was developed, which allowed for the screening of genotypic differences in rice seedlings for this trait. Germanium has previously been used to screen rice mutants, which identified the first silicon transporter in rice *Lsi1* [3], and it was also subsequently used to identify *Lsi2* in rice [4]. A study by Sparks *et al*., (2010) demonstrated that the subcellular localisation of silicon and germanium in the roots and shoots of two grasses is the same, indicating that germanium is a good biological analogue of silicon [15]. Therefore the germanium induced lesion screening method was used to identify QTLs in two rice populations, and subsequent analysis was performed to see if any of the identified loci co-localised with previously identified silicon QTLs. Germanium induced QTLs were also compared to the locations of previously identified arsenic QTLs, as arsenite, an inorganic arsenic species, is an analogue of silicon.

The high levels of heritability for all germanium induced lesions within the Bala x Azucena mapping population (greater than 75%) denoted that the variation of germanium induced lesions observed in the RILs was under strong genetic control (Table 1). The variation explained by genotypic differences in the genome wide association mapping was also large (ranging from 60.3% to 66.3%). A large number of QTLs and significantly associated SNPs were detected in the Bala x Azucena mapping population and the RDP1 association mapping population for germanium induced lesions, indicating that the trait is associated with more than one loci. There are two loci which were detected in both the Bala x Azucena mapping population and in the RDP1. These were the QTLs at the top of chromosome 1 and the lower part of chromosome 3 (Fig 2). As these were detected in more than one analysis it makes these interesting loci for further investigation. As well as co-localisation of QTLs between the two experiments conducted here it was also observed that the QTLs for germanium induced lesions also co-localised with previously identified QTLs for husk silicon [27,44], grain arsenic QTLs [21], and QTLs for grain organic arsenic content [23] (S3 Table). Of the 17 significant SNPs detected in the GWA mapping using all the accessions, 6 SNPs (4 loci) co-localise with previously detected QTLs for rice silicon or arsenic (S3 Table). Of the 54 significant SNPs detected in the analysis of the subpopulations, 7 co-localise with previously detected QTLs for arsenic or silicon concentration in rice plants (S5 Table).

Three genes have been identified in rice as being in silicon transporters: *Lsi1*, *Lsi2*, and *Lsi6* [3–5]. In addition, recent research indicates that there may be other genes (as yet not identified) which are involved in silicon transport in rice [6]. There were no QTLs or significant SNPs identified in this study near *Lsi1* (LOC_Os02g51110) on chromosome 2 or *Lsi6* (LOC_Os06g12310) on chromosome 6. A single significant SNP identified in the GWA mapping within the *tropical japonicas* was detected within 200 kb of *Lsi2* (LOC_Os03g01700) on chromosome 3. The lack of identified QTLs and SNPs within 200 kb of these known candidate genes was surprising. It is unlikely that the lack of an association is due to insufficient SNP coverage as there are between 5–10 SNPs within 200 kb of each of these genes. There are a number of possible explanations: (1) that there is no polymorphism for these genes, or (2) if there is polymorphism for these genes, that the germanium induced lesion trait is not an adequate tool to detect these polymorphisms. To address the second point an experiment was conducted to determine if the germanium lesion trait is related to the concentration of germanium in the cultivar. This experiment shows that there is about twofold difference of germanium accumulation within the selected genotypes. There was a positive relationship between germanium induced lesions and germanium concentration in the plants (Fig 4), however it only explained 22.8% of the variation. This indicates that the presence of germanium induced lesions is a result of not only the concentration of germanium in the plants (otherwise a relationship explaining a greater percentage of the variation would have been expected), but also of other factors, which could include genotypic differences in sensitivity to germanium. For example, there are two cultivars that have a tissue concentration of ~12 mg/g however the percentage of germanium induced lesions are ~5% for one cultivar and ~60% for the other. Thus, it is assumed that some of the QTLs detected here are not germanium uptake QTLs but also tissue sensitivity QTLs. It is expected that the uptake QTLs would be co-localised with the previously identified silicon QTLs, but the tissue sensitivity QTLs would not be expected to be co-localised with these QTLs.

A number of good candidate genes were identified, with the two most promising genes being homologues of a gene previously identified as being involved in silicon uptake and distribution in rice [3–5]. The candidate gene on chromosome 1 (Os01g02190) had very high homology with *Lsi6* (*Lsi6* is also an analogue of *Lsi1*). *Lsi6* is a gene that regulates silicon distribution in rice shoots [5]. However, when exploring the available expression data (http://ricexpro.dna.affrc.go.jp/) on Os01g02190, it appears that this gene is only expressed in the anthers. The candidate gene on chromosome 10 (Os10g31040) has high homology to *Lsi2*, a gene which is categorised as an efflux transporter of silicon in rice [3,4]. In addition to these genes with homology to known silicon transporters, a number of candidate genes were annotated as ABC transporters. An ABC transporter (OsABCC1) has recently been shown to be involved in the transport of arsenic in rice grains [45]. Further studies are needed to elucidate if these genes have a role in the uptake and distribution of germanium (and silicon) in rice plants.

In conclusion, this study demonstrates a simple screen of germanium phenotypes, using germanic acid in the nutrient solution to screen for natural variation in germanium induced lesions. This method has one shortcoming: the germanium toxicity lesion in response to germanium accumulation indicates some difference in tissue sensitivity to germanium between genotypes. So long as this is taken into account when interpreting the data, the method appears valuable in discriminating the genotypes according to germanium accumulation in plants, and can be used for genetic mapping. In this study a number of good candidate genes were identified, that are deemed worthy of future study to identify their potential role in germanium and silicon accumulation.

## Supporting Information

S1 Fig Genotypic difference of Ge induced lesions in Azucena (A) 5% lesions and Bala (B) 60% lesions when exposed to 15 μM Ge for 3 days. (TIFF)

S2 Fig Frequency distribution of the germanium induced lesion trait in the Bala x Azucena F_6_ population on day 4 (A), day 5 (B), day 6 (C), day 7 (D), and day 8 (E). (TIF)

S3 Fig Frequency distribution of the germanium induced lesion trait in the rice diversity association population on day 4 (A), day 5 (B), and day 6 (C) (TIFF)

S4 Fig Distribution of the germanium induced lesion trait in four subpopulations from the rice diversity population on day 4 (A), day 5 (B), and day 6 (C). (TIFF)

S1 Table Quantitative trait loci for germanium induced lesions (%) within the Azucena x Bala F_6_ mapping population. Putative QTLs are in italics on the table.
(DOCX)

S2 Table Significant SNPs (P<0.0001; MAF>5%) associated with the germanium induced lesion phenotype on days 4, 5, and 6 identified in the GWA mapping using all cultivars. (DOCX)

S3 Table Significant SNPs (P<0.0001; MAF>5%) associated with the germanium induced lesion phenotype on days 4, 5, and 6 identified in the GWA mapping using cultivars from each of the 4 rice subgroups (*aus (*AUS), *indica* (*IND*), *temperate japonica* (*TEJ*) and *tropical japonica* (*TRJ*). (DOCX)

S4 Table Summary of QTL and GWA mapping association positions and number of positional candidate genes for each loci. (DOCX)

S5 Table Co-localisation of SNPs significantly associated with germanium induced lesions and QTLs for rice arsenic content and silicon content. (DOCX)

## References

[1] Yoshida S. Fundamental of rice crop science Philippines: The International Rice Research Institute; 1981.

[2] Detmann KC, Araujo WL, Martins SCV, Sanglard LMVP, Reis JV, Detmann E, et al Silicon nutrition increases grain yield, which, in turn, exerts a feed-forward stimulatin of photosynthetic rates via enhanced mesophyll conductance and alters primary metabolism in rice. New Phytologist. 2012;196:752–62. 10.1111/j.1469-8137.2012.04299.x 22994889

[3] Ma JF, Tamai K, Yamaji N, Mitani N, Konishi S, Katsuhara M, et al A silicon transporter in rice. Nature. 2006;440:688–91. 16572174 10.1038/nature04590

[4] Ma JF, Yamaji N, Mitani N, Tamai K, Konishi S, Fujiwara T, et al An efflux transporter of silicon in rice. Nature. 2007;448:209–12 17625566 10.1038/nature05964

[5] Yamaji N, Mitani N, Ma JF. A transporter regulating silicon distribution in rice shoot. The Plant Cell. 2008;20:1381–89. 10.1105/tpc.108.059311 18515498 PMC2438455

[6] Sakurai G, Satake A, Yamaji N, Mitani-Ueno N, Yokozawa M, Feugier FG, et al In silico simulation modelling reveals the importance of the casparian strip for efficient silicon uptake in rice roots. Plant Cell Physiology. 2015;56(4):631–39. 10.1093/pcp/pcv017 25673476

[7] Abedin MJ, Cresser MS, Meharg AA, Feldmann J, Cotter-Howells J. Arsenic accumulation and metabolism in rice (*Oryza sativa* L.). Environmental Science and Technology. 2002;36:962–68. 11918027 10.1021/es0101678

[8] Ma JF, Yamaji N, Mitani N, Xu XY, Su YH, McGrath SP, et al Transporters of arsenite in rice and their role in arsenic accumulation in rice grain. Proc Natl Acad Sci. 2008; 105:9931−35. 10.1073/pnas.0802361105 18626020 PMC2481375

[9] Xu XY, McGrath SP, Meharg AA, Zhao FJ. Growing rice aerobically markedly decreases arsenic accumulation. Environmental Science and Technology.2008;42:5574–79. 18754478 10.1021/es800324u

[10] Zhao FJ, Ma JF, Meharg AA, McGrath SP. Arsenic uptake and metabolism in plants. New Phytologist. 2009;181:777–94. 10.1111/j.1469-8137.2008.02716.x 19207683

[11] Takahashi E, Syo S, Miyake Y. Effect of germanium on the growth of plants with special reference to the silicon nutrition (Part 1). Journal of the Science of Soil and Manure, Japan. 1976;47:183–90.

[12] Takahashi E, Syo S, Miyake Y. Effect of germanium on the growth of plants with special reference to the silicon nutrition (Part 2). Journal of the Science of Soil and Manure, Japan 1976;47:191–97.

[13] Rains DW, Epstein E, Zasoski RJ, Aslam M. Active silicon uptake by wheat. Plant and Soil. 2006;280:223–28.

[14] Mitani N, Ma JF. Uptake system of silicon in different plant species. Journal of Experimental Botany. 2005;414:1255–61. 10.1093/jxb/eri121 15753109

[15] Sparks JP, Chandra S, Derry LA, Parthasarathy MV, Daugherty CS, Griffin R. Subcellular localization of silicon and germanium in grass root and leaf tissues by SIMS: evidence for differential and active transport. Biogeochemistry. 2010;104:237–49.

[16] Carey AM, Norton GJ, Deacon C, Scheckel KG, Lombi E, Punshon T, et al Phloem transport of arsenic species from flag leaf to grain during grain filling. New Phytologist. 2011;192:87–98. 10.1111/j.1469-8137.2011.03789.x 21658183 PMC3932528

[17] Hayes JE, Pallotta M, Baumann U, Berger B, Langridge P, Sutton T. Germanium as a tool to dissect boron toxicity effects in barley and wheat. Functional Plant Biology. 2013;40:618–27. 10.1071/FP12329 32481135

[18] Norton GJ, Deacon CM, Xiong L, Huang S, Meharg AA, Price AH. Genetic mapping of the rice ionome in leaves and grain: Identification of QTLs for 17 elements including arsenic, cadmium, iron and selenium. Plant and Soil. 2010;329:139–53.

[19] Norton GJ, Duan GL, Lei M, Zhu YG, Meharg AA, Price AH. Identification of quantitative trait loci for rice grain element composition on an arsenic impacted soil: Influence of flowering time on genetic loci. Annals of Applied Biology. 2012;161:46–56.

[20] Zhang J, Zhu YG, Zeng DL, Cheng WD, Qian Q, Duan GL. Mapping quantitative trait loci associated with arsenic accumulation in rice (*Oryza sativa*). New Phytologist. 2008;177:350–55. 17995916 10.1111/j.1469-8137.2007.02267.x

[21] Zhang M, Pinson SRM, Tarpley L, Huang XY, Lahner B, Yakubova E, et al Mapping and validation of quantitative trait loci associated with concentration of 16 elements in unmilled rice grain. Theoretical and Applied Genetics. 2014;127:137–65. 24231918 10.1007/s00122-013-2207-5 PMC4544570

[22] Norton GJ, Douglas A, Lahner B, Yakubova E, Guerinot ML, Pinson SRM, et al Genome Wide Association Mapping of Grain Arsenic, Copper, Molybdenum and Zinc in Rice (*Oryza sativa* L) Grown at Four International Field Sites. Plos One. 2014;9:2 10.1371/journal.pone.0089685 PMC3934919 24586963

[23] Kuramata M, Abe T, Kawasaki A, Ebana K, Shibaya T, Yano M, et al Genetic diversity of arsenic accumulation in rice and QTL analysis of methylated arsenic in rice grains. Rice. 2013;6:3 10.1186/1939-8433-6-3 24280235 PMC5394917

[24] Jia Y, Huang H, Sun GX, Zhao FJ, Zhu YG. Pathways and relative contributions to arsenic volatilization from rice plants and paddy soil. Envrionmnetal Science and Technology. 2012;46:8090–96. 10.1021/es300499a 22724924

[25] Lomax C, Lui WJ, Wu L, Xue K, Xiong J, Zhou J, et al Methylated arsenic species in plants originate from soil microorganisms. New Phytologist. 2012;193:665–72. 10.1111/j.1469-8137.2011.03956.x 22098145

[26] Li RY, Ago Y, Liu WJ, Mitani N, Feldmann J, McGrath SP, et al The Rice Aquaporin Lsi1 Mediates Uptake of Methylated Arsenic Species. Plant Physiology. 2009;150:2071–80. 10.1104/pp.109.140350 19542298 PMC2719116

[27] Dai WM, Zhang KQ, Duan BW, Zheng KL, Zhuang JY, Cai R. Genetic dissection of silicon content in different organs of rice. Crop Science. 2005;45:1345–52.

[28] Wu QS, Wan XY, Su N, Cheng ZJ, Wang JK, Lei CL, et al Genetic dissection of silicon uptake ability in rice (*Oryza sativa* L.). Plant Science. 2006;171:441–48. 10.1016/j.plantsci.2006.05.001 25193641

[29] Bryant R, Procter A, Hawkridge M, Jackson A, Yeater K, Counce P, et al Genetic variation and association mapping of silica concentration in rice hulls using a germplasm collection. Genetica. 2011;139:1383–98. 10.1007/s10709-012-9637-x 22403009

[30] Yoshida S, Forno DA, Cock JH, Gomez KA. Laboratory Manual for the Physiological Studies of Rice Third Edition Philippines: THE INTERNATIONAL RICE RESEARCH INSTITURE; 1976.

[31] Price AH, Steele KA, Moore BJ, Barraclough PP, Clark LJ. A combined RFLP and AFLP linkage map of upland rice (*Oryza sativa* L.) used to identify QTLs for root-penetration ability. Theoretical and Applied Genetics. 2000;100:49–56.

[32] Price AH, Steele KA, Moore BJ, Jones RGW. Upland rice grown in soil-filled chambers and exposed to contrasting water-deficit regimes. II. Mapping quantitative trait loci for root morphology and distribution. Field Crops Research. 2002;76:25–43.

[33] Norton GJ, Price AH. Mapping of quantitative trait loci for seminal root morphology and gravitropic response in rice. Euphytica. 2009;166:229–37.

[34] Basten JC, Weir SB, Zeng BZ. QTL Cartographer version 1.15. Department of Statistics, North Carolina State University 2001.

[35] Wang DL, Zhu J, Li ZK, Paterson AH. Mapping QTLs with epistatic effects and QTL x environment interactions by mixed linear model approaches. Theoretical Applied Genetics. 1999;99:1255–1264.

[36] Wang DL, Zhu J, Li ZK, Paterson AH. User manual for QTLMapper version 1.0 Texas A&M Univ., College Station, TX 1999.

[37] Tung CW, Zhao K, Wright MH, Ali ML, Jung J, Kimball J, et al Development of a research platform for dissecting phenotype–genotype associations in rice (*Oryza* spp.). Rice. 2010;3:205–17.

[38] Zhao K, Wright M, Kimball J, Eizenga G, McClung A, Kovach M. et al Genomic diversity and introgression in *O*. *sativa* reveal the impact of domestication and breeding on the rice genome. PLoS One. 2010;5:5. 10.1371/journal.pone.0010780 PMC2875394 20520727

[39] Eizenga GC, Ali ML, Bryant RJ, Yeater KM, McClung AM, McCouch SR. Registration of the ‘Rice Diversity Panel 1’ for genome-wide association studies. Journal of Plant Registrations. 2014;8:109–16.

[40] Zhao K, Tung CW, Eizenga GC, Wright MH, Ali ML, Price AH, et al Genome-wide association mapping reveals a rich genetic architecture of complex traits in *Oryza sativa* . Nature Communications. 2011;2:467 10.1038/ncomms1467 21915109 PMC3195253

[41] Yu J, Pressoir G, Briggs WH, Vroh Bi I, Yamasaki M, Doebley JF, et al A unified mixed-model method for association mapping that accounts for multiple levels of relatedness. Nat Genet. 2006;38:203–8. 16380716 10.1038/ng1702

[42] Sun G, Williams PN, Zhu YG, Deacon C, Carey AM, Raab A, et al Survey of arsenic and its speciation in rice products such as breakfast cereals, rice crackers and Japanese rice condiments. Environment International. 2009;35:473–75. 10.1016/j.envint.2008.07.020 18775567

[43] Price AH. Believe it or not, QTLs are accurate! Trends in Plant Science. 2006;11 **:** 213–16. 16617032 10.1016/j.tplants.2006.03.006

[44] Dai WM, Zhang KQ, Wu JR, Wang L, Duan BW, Zheng KL, et al Validating a segment on the short arm of chromosome 6 responsible for genetic variation in the hull silicon content and yield traits of rice. Euphytica. 2008;160:317–24.

[45] Song WY, Yamaki T, Yamaji N, Ko D, Jung KH, Fujii-Kashino M, et al A rice ABC transporter, OsABCC1, reduces arsenic accumulation in the grain. Proc Natl Acad Sci. 2014; 111:15699–704. 10.1073/pnas.1414968111 25331872 PMC4226097

