# Supplementary figures and images for: Biallelic and Genome Wide Association Mapping of Germanium Tolerant Loci in Rice (Oryza sativa L.)
Source: PLoS One. 2015 Sep 10;10(9):e0137577. doi: 10.1371/journal.pone.0137577 (PMC4565582; doi:10.1371/journal.pone.0137577)

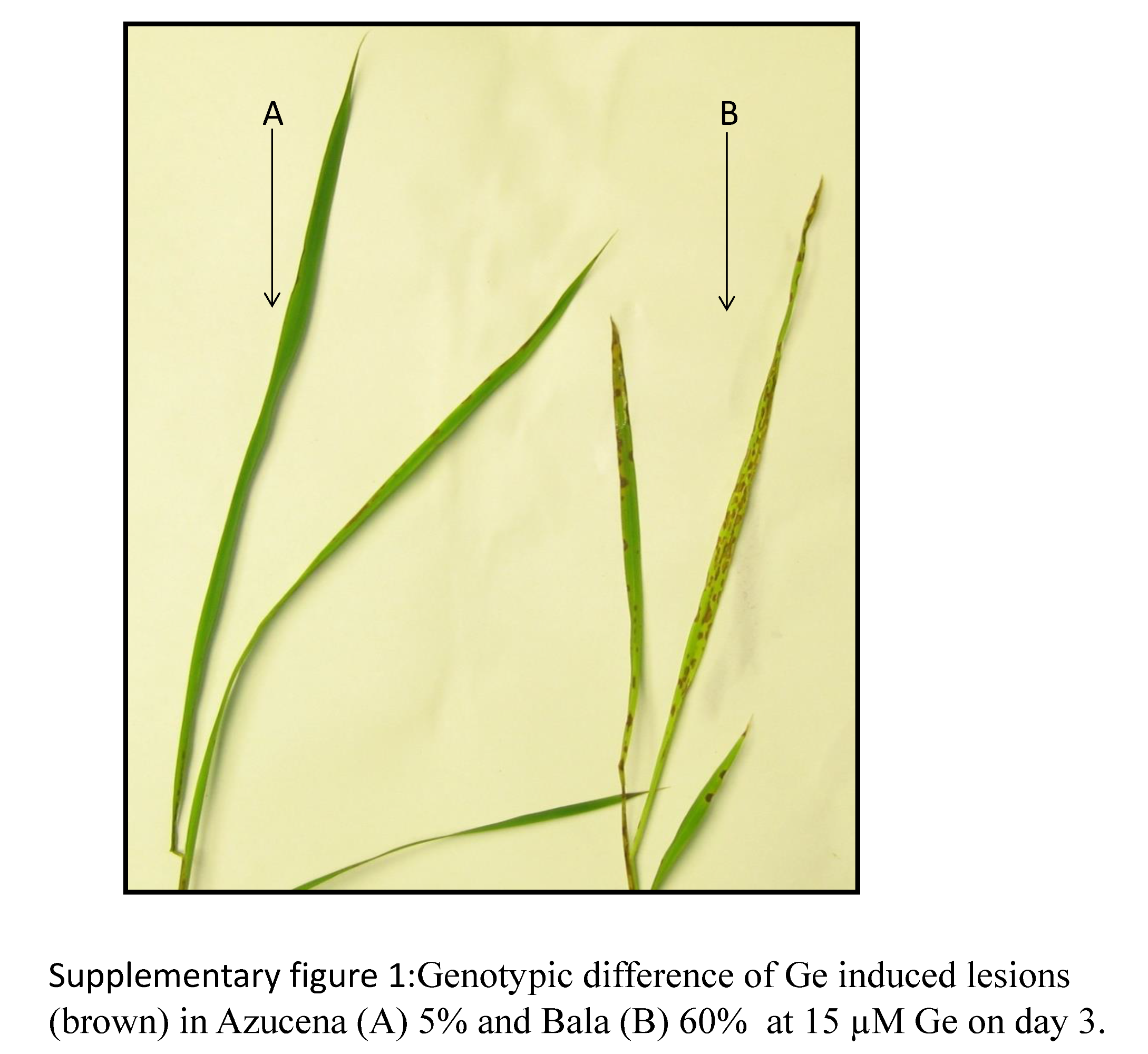

Supplement: S1 Fig — (TIFF) [file pone.0137577.s001.tiff]

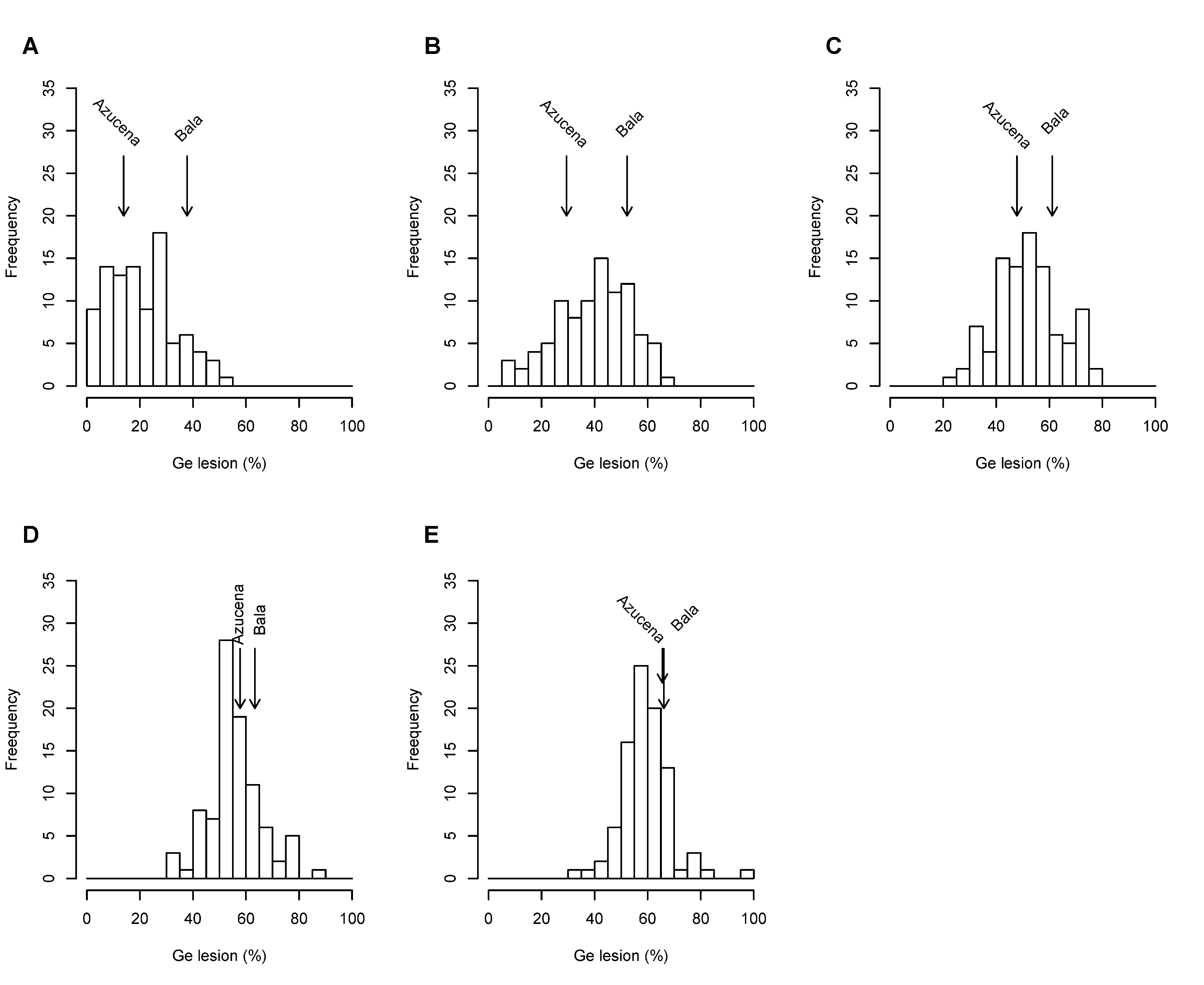

Supplement: S2 Fig — (TIF) [file pone.0137577.s002.tif]

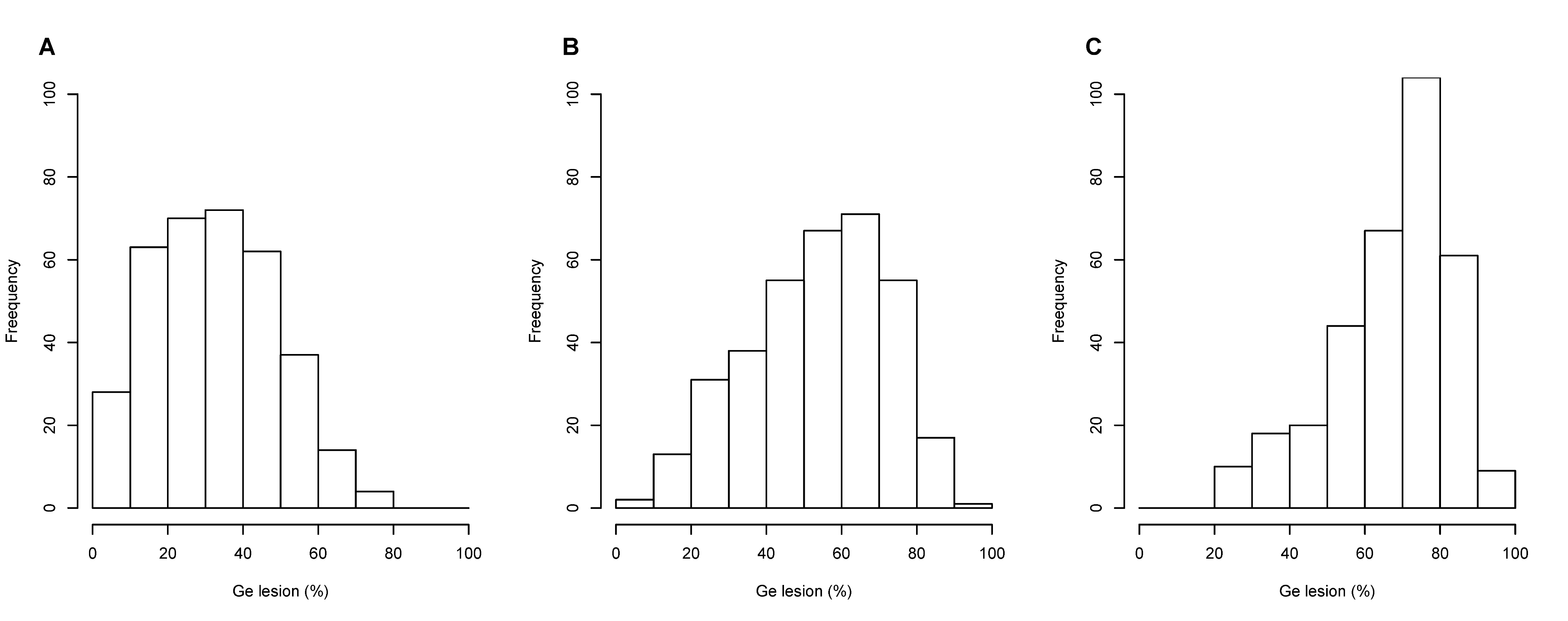

Supplement: S3 Fig — (TIFF) [file pone.0137577.s003.tiff]

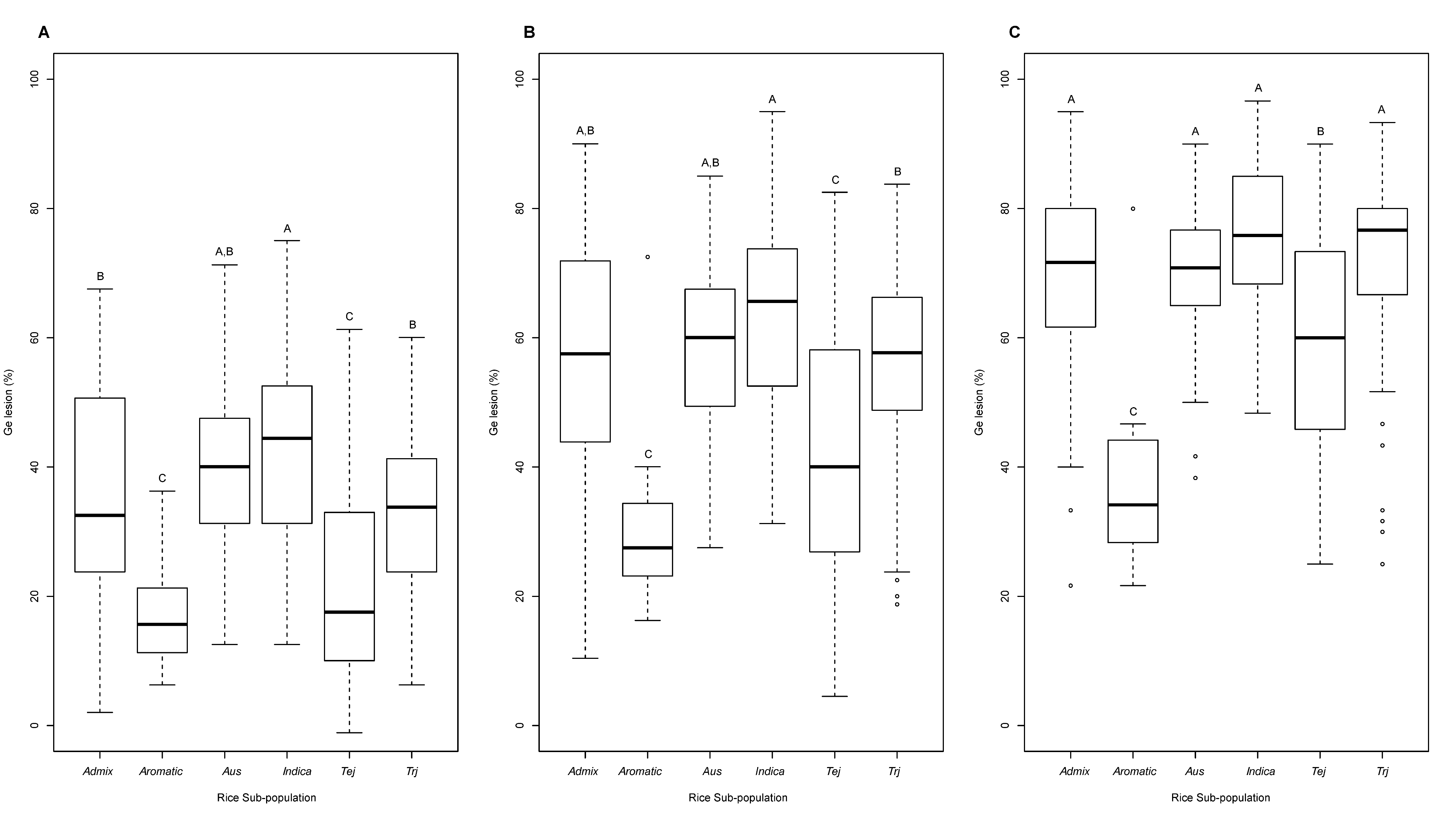

Supplement: S4 Fig — (TIFF) [file pone.0137577.s004.tiff]
