# Supplementary material for: Biallelic and Genome Wide Association Mapping of Germanium Tolerant Loci in Rice (Oryza sativa L.)
Source: PLoS One. 2015 Sep 10;10(9):e0137577. doi: 10.1371/journal.pone.0137577 (PMC4565582; doi:10.1371/journal.pone.0137577)
Supplement: S1 Table — Putative QTLs are in italics on the table. (DOCX) [file pone.0137577.s005.docx]

Supplementary Table 1. Quantitative trait loci for germanium induced lesions (%) within the Azucena x Bala F_6_ mapping population. Putative QTLs are in italics on the table.

| Trait | QTL name | Chromosome | QTL position in cM | | QTL peak (Mb) | LOD | R^2^ | Donor of Positive allele |
| --- | --- | --- | --- | --- | --- | --- | --- | --- |
|  |  |  | Above (-) or  below (+)  nearest marker | From the top of the linkage group |  |  |  |  |
| Ge4 | Ge4.1 | 2 | G45-32 | 60 | 13.02 | 5.3 | 13 | Bala |
| Ge4 | Ge4.2 | 3 | RG745-24 | 45 | 8.33 | 6.9 | 25 | Bala |
| Ge4 | Ge4.3 | 3 | G164-13 | 160 | 32.00 | 4.6 | 14 | Bala |
| Ge5 | Ge5.1 | 2 | G45-37 | 55 | 11.58 | 7.5 | 26 | Bala |
| *Ge5* | *Ge5.3* | *3* | *RG191-8* | *18* | *3.84* | *3.0* | *8* | *Bala* |
| *Ge5* | *Ge5.4* | *3* | *G164-5* | *168* | *33.32* | *3.3* | *10* | *Bala* |
| *Ge5* | *Ge5.5* | *8* | *R662-0.5* | *144* | *27* | *3.0* | *7* | *Bala* |
| Ge6 | Ge6.1 | 1 | RM7278-6 | 2 | 1.01 | 5.3 | 14 | Bala |
| Ge6 | Ge6.2 | 2 | G45-32.7 | 60 | 13.02 | 9.0 | 19 | Bala |
| Ge6 | Ge6.3 | 3 | G164-3 | 170 | 33.65 | 7.7 | 17 | Bala |
| *Ge6* | *Ge6.4* | *11* | *AB1101-4* | *84* | *21.22* | *3.2* | *5* | *Azucena* |
| Ge7 | Ge7.1 | 1 | RM7278-8 | 0 | 0.75 | 5.5 | 15 | Bala |
| Ge7 | Ge7.2 | 2 | G45-32 | 60 | 13.02 | 5.6 | 12 | Bala |
| Ge7 | Ge7.3 | 3 | G164-7 | 166 | 32.99 | 6.7 | 21 | Bala |
| Ge7 | Ge7.5 | 5 | C624-12 | 62 | 16.43 | 3.9 | 9 | Azucena |
| *Ge7* | *Ge7.6* | *11* | *AB1101-4* | *85* | *21.32* | *2.9* | *5* | *Azucena* |
| *Ge7* | *Ge7.7* | *12* | *G124-2* | *25* | *3.57* | *3.0* | *6* | *Azucena* |
| Ge8 | Ge8.1 | 1 | RM7278-8 | 0 | 0.76 | 6.2 | 18 | Bala |
| Ge8 | Ge8.2 | 2 | G45-35 | 57 | 12.16 | 3.6 | 10 | Bala |
| Ge8 | Ge8.3 | 3 | G164-3 | 170 | 33.65 | 5.2 | 17 | Bala |
| *Ge8* | *Ge8.4* | *4* | *RM252-21* | *50* | *21.24* | *3.0* | *13* | *Azucena* |
| Ge8 | Ge8.5 | 5 | C624-12 | 62 | 16.43 | 5.1 | 13 | Azucena |
