# Supplementary material for: Biallelic and Genome Wide Association Mapping of Germanium Tolerant Loci in Rice (Oryza sativa L.)
Source: PLoS One. 2015 Sep 10;10(9):e0137577. doi: 10.1371/journal.pone.0137577 (PMC4565582; doi:10.1371/journal.pone.0137577)
Supplement: S2 Table — (DOCX) [file pone.0137577.s006.docx]

Supplementary table 2. Significant SNPs (P<0.0001; MAF>5%) associated with the germanium induced lesion phenotype on days 4, 5, and 6 identified in the GWA mapping using all cultivars.

| Trait | Analysis | SNP ID | P-Value | Chromosome | Mbp | MAF (%) |
| --- | --- | --- | --- | --- | --- | --- |
| Ge6 | ALL | id1013420 | 4.07E-05 | 1 | 23.37 | 41.7 |
| Ge5 | ALL | id1015789 | 7.27E-06 | 1 | 27.10 | 23.4 |
| Ge6 | ALL | id1015789 | 2.57E-05 | 1 | 27.10 | 23.4 |
| Ge5 | ALL | id1015794 | 7.27E-06 | 1 | 27.10 | 23.4 |
| Ge6 | ALL | id1015794 | 2.57E-05 | 1 | 27.10 | 23.4 |
| Ge6 | ALL | id2000088 | 4.32E-05 | 2 | 0.19 | 38.4 |
| Ge5 | ALL | id3012850 | 1.32E-05 | 3 | 28.03 | 17.0 |
| Ge6 | ALL | id3012850 | 4.15E-05 | 3 | 28.03 | 17.0 |
| Ge4 | ALL | id3012850 | 6.16E-05 | 3 | 28.03 | 17.0 |
| Ge6 | ALL | id3015629 | 3.31E-05 | 3 | 32.47 | 44.3 |
| Ge6 | ALL | id3015632 | 4.07E-05 | 3 | 32.47 | 37.3 |
| Ge5 | ALL | id3017913 | 7.74E-05 | 3 | 35.82 | 23.1 |
| Ge5 | ALL | id3018359 | 3.16E-05 | 3 | 36.13 | 17.8 |
| Ge5 | ALL | id6003502 | 2.13E-05 | 6 | 5.21 | 43.2 |
| Ge4 | ALL | id6003502 | 8.12E-05 | 6 | 5.21 | 43.2 |
| Ge5 | ALL | id10004574 | 6.02E-05 | 10 | 16.41 | 32.8 |
| Ge5 | ALL | id10004705 | 7.62E-05 | 10 | 16.87 | 28.0 |
