# Supplementary material for: Biallelic and Genome Wide Association Mapping of Germanium Tolerant Loci in Rice (Oryza sativa L.)
Source: PLoS One. 2015 Sep 10;10(9):e0137577. doi: 10.1371/journal.pone.0137577 (PMC4565582; doi:10.1371/journal.pone.0137577)
Supplement: S3 Table — (DOCX) [file pone.0137577.s007.docx]

Supplementary table 3. Significant SNPs (P<0.0001; MAF>5%) associated with the germanium induced lesion phenotype on days 4, 5, and 6 identified in the GWA mapping using cultivars from each of the 4 rice subgroups (*aus (*AUS*), indica* (*IND*)*, temperate japonica* (*TEJ*) and *tropical japonica* (*TRJ*).

| Trait | Analysis | SNP ID | P-Value | Chromosome | Mbp | MAF (%) |
| --- | --- | --- | --- | --- | --- | --- |
| Ge4 | *TRJ* | id1001153 | 7.07E-05 | 1 | 1.4 | 35.1 |
| Ge5 | *IND* | id1006990 | 9.79E-05 | 1 | 9.32 | 45.2 |
| Ge5 | *IND* | id2006855 | 1.67E-05 | 2 | 17.21 | 44.1 |
| Ge6 | *TRJ* | id3000877 | 8.68E-06 | 3 | 1.62 | 6.3 |
| Ge4 | *TEJ* | id3006227 | 4.06E-05 | 3 | 12.26 | 35.5 |
| Ge4 | *TEJ* | id3006236 | 3.95E-05 | 3 | 12.26 | 36.0 |
| Ge6 | *AUS* | id4004135 | 4.88E-05 | 4 | 14.08 | 9.3 |
| Ge6 | *AUS* | id4004154 | 4.88E-05 | 4 | 14.09 | 9.3 |
| Ge4 | *AUS* | id4005078 | 7.17E-05 | 4 | 17.43 | 21.4 |
| Ge5 | *AUS* | id4005078 | 7.94E-05 | 4 | 17.43 | 21.4 |
| Ge5 | *TRJ* | id5008807 | 3.37E-05 | 5 | 20.88 | 13.6 |
| Ge6 | *TRJ* | id5008807 | 5.86E-05 | 5 | 20.88 | 13.5 |
| Ge4 | *TEJ* | id6005251 | 4.2E-05 | 6 | 8.09 | 32.9 |
| Ge6 | *TRJ* | ud6000585 | 9.3E-05 | 6 | 13.52 | 8.9 |
| Ge6 | *TRJ* | ud6000586 | 5.2E-05 | 6 | 13.56 | 9.0 |
| Ge6 | *TRJ* | id6008114 | 9.3E-05 | 6 | 13.58 | 8.9 |
| Ge6 | *TRJ* | id6008142 | 9.3E-05 | 6 | 13.60 | 8.9 |
| Ge6 | *TRJ* | id6008193 | 9.3E-05 | 6 | 13.67 | 8.9 |
| Ge6 | *TRJ* | id6008210 | 9.3E-05 | 6 | 13.70 | 8.9 |
| Ge6 | *TRJ* | id6008219 | 9.3E-05 | 6 | 13.70 | 8.9 |
| Ge6 | *TRJ* | id6008273 | 9.3E-05 | 6 | 13.77 | 8.9 |
| Ge6 | *TRJ* | id6008275 | 9.3E-05 | 6 | 13.77 | 8.9 |
| Ge6 | *TRJ* | id6008302 | 9.3E-05 | 6 | 13.80 | 8.9 |
| Ge6 | *TRJ* | id6008575 | 3.12E-05 | 6 | 14.27 | 12.3 |
| Ge5 | *TRJ* | id6008730 | 5.96E-05 | 6 | 14.53 | 12.5 |
| Ge4 | *TRJ* | id6008763 | 9.55E-05 | 6 | 14.66 | 14.1 |
| Ge5 | *TRJ* | id6008763 | 8.59E-05 | 6 | 14.66 | 14.5 |
| Ge6 | *TRJ* | wd6002100 | 3.02E-05 | 6 | 15.91 | 12.8 |
| Ge4 | *TRJ* | id6009266 | 9.43E-05 | 6 | 16.26 | 16.4 |
| Ge6 | *TRJ* | id6009292 | 3.02E-05 | 6 | 16.32 | 12.8 |
| Ge6 | *TRJ* | wd7003219 | 2.43E-05 | 7 | 25.12 | 9.0 |
| Ge4 | *TRJ* | id7005543 | 3.1E-05 | 7 | 27.79 | 25.3 |
| Ge6 | *TRJ* | id8000422 | 3.85E-05 | 8 | 1.63 | 11.1 |
| Ge6 | *TRJ* | id8000432 | 3.85E-05 | 8 | 1.64 | 5.1 |
| Ge6 | *TRJ* | id8000436 | 3.77E-05 | 8 | 1.64 | 11.4 |
| Ge6 | *TRJ* | id8000442 | 7.46E-06 | 8 | 1.64 | 11.5 |
| Ge6 | *TRJ* | id8002076 | 4.5E-06 | 8 | 6.04 | 6.4 |
| Ge4 | *TRJ* | id10004975 | 2.51E-05 | 10 | 17.34 | 21.0 |
| Ge5 | *TRJ* | id10004975 | 7.4E-06 | 10 | 17.34 | 21.0 |
| Ge6 | *TRJ* | id10004975 | 1.34E-05 | 10 | 17.34 | 21.0 |
| Ge5 | *TRJ* | id10004985 | 5.66E-05 | 10 | 17.38 | 6.1 |
| Ge5 | *TRJ* | id10005002 | 6.68E-05 | 10 | 17.41 | 6.2 |
| Ge6 | *TRJ* | id11005253 | 7.97E-05 | 11 | 15.45 | 6.3 |
| Ge5 | *TRJ* | id11009869 | 2.94E-05 | 11 | 24.77 | 12.5 |
| Ge6 | *TRJ* | id11010268 | 9.81E-06 | 11 | 25.58 | 7.6 |
| Ge6 | *TRJ* | id11010272 | 1.24E-05 | 11 | 25.58 | 7.9 |
| Ge4 | *TRJ* | id12009421 | 4.78E-06 | 12 | 25.86 | 39.0 |
| Ge4 | *TRJ* | id12009451 | 5.95E-05 | 12 | 25.87 | 42.7 |
| Ge4 | *TRJ* | id12009470 | 8.69E-06 | 12 | 25.89 | 43.9 |
| Ge6 | *TRJ* | id12009470 | 1.78E-05 | 12 | 25.89 | 43.9 |
| Ge4 | *TRJ* | id12009504 | 1.6E-05 | 12 | 25.94 | 43.2 |
| Ge6 | *TRJ* | id12009504 | 1.78E-05 | 12 | 25.94 | 43.2 |
| Ge4 | *TRJ* | id12009654 | 1.58E-05 | 12 | 26.10 | 40.0 |
| Ge5 | *TRJ* | id12009654 | 7.87E-05 | 12 | 26.10 | 40.0 |
