# Supplementary material for: Biallelic and Genome Wide Association Mapping of Germanium Tolerant Loci in Rice (Oryza sativa L.)
Source: PLoS One. 2015 Sep 10;10(9):e0137577. doi: 10.1371/journal.pone.0137577 (PMC4565582; doi:10.1371/journal.pone.0137577)
Supplement: S4 Table — (DOCX) [file pone.0137577.s008.docx]

Supplementary table 4. Summary of QTL and GWA mapping association positions and number of positional candidate genes for each loci.

| **Mapping experiment** | **Trait name** | **Chromosome** | **Genomic location (Mbp)** | **No. of genes** |
| --- | --- | --- | --- | --- |
| QTL | Ge7 & Ge8 | 1 | 0.1-1.41 | 179 |
| QTL | Ge6 | 1 | 0.8-2.1 | 184 |
| QTL | Ge5 | 2 | 10.12-13.3 | 291 |
| QTL | Ge4, Ge5, Ge6, Ge7 & Ge8 | 2 | 11.55-14.47 | 243 |
| QTL | Ge4 | 3 | 7.5-9.1 | 217 |
| QTL | Ge5 | 3 | 2.83-4.84 | 312 |
| QTL | Ge4, Ge5, Ge6, Ge7 & Ge8 | 3 | 32.16-33.81 | 265 |
| QTL | Ge8 | 4 | 20.33-22.14 | 222 |
| QTL | Ge7 & Ge8 | 5 | 15.57-17.28 | 160 |
| QTL | Ge5 | 8 | 26.65-28.5 | 222 |
| QTL | Ge6 & Ge7 | 11 | 20.37-22.06 | 201 |
| QTL | Ge7 | 12 | 2.52-4.61 | 260 |
| GWAS All | Ge6 | 1 | 23.17-23.57 | 49 |
| GWAS All | Ge5 & Ge6 | 1 | 26.9-27.3 | 53 |
| GWAS All | Ge6 | 2 | 0.00-0.39 | 58 |
| GWAS All | Ge4, Ge5, Ge6 | 3 | 27.83-28.23 | 57 |
| GWAS All | Ge6 | 3 | 32.27-32.67 | 69 |
| GWAS All | Ge5 | 3 | 35.62-36.02 | 66 |
| GWAS All | Ge5 | 3 | 35.93-36.33 | 56 |
| GWAS All | Ge4 & Ge5 | 6 | 5.01-5.41 | 62 |
| GWAS All | Ge5 | 10 | 16.21-16.61 | 36 |
| GWAS All | Ge5 | 10 | 16.67-17.07 | 50 |
| GWAS TRJ | Ge4 | 1 | 1.2-1.6 | 58 |
| GWAS IND | Ge5 | 1 | 9.12-9.52 | 67 |
| GWAS IND | Ge5 | 2 | 17.01-17.41 | 49 |
| GWASTRJ | Ge6 | 3 | 1.42-1.82 | 83 |
| GWAS TEJ | Ge4 | 3 | 12.06-12.46 | 57 |
| GWAS AUS | Ge6 | 4 | 13.88-14.28 | 37 |
| GWAS AUS | Ge4 & Ge5 | 4 | 17.23-17.63 | 21 |
| GWAS TRJ | Ge5 & Ge6 | 5 | 20.68-21.08 | 71 |
| GWAS TEJ | Ge4 | 6 | 7.89-8.29 | 42 |
| GWAS TRJ | Ge6 | 6 | 13.32-13.72 | 48 |
| GWAS TRJ | Ge6 | 6 | 13.57-13.97 | 43 |
| GWAS TRJ | Ge6 | 6 | 13.6-14 | 48 |
| GWAS TRJ | Ge6 | 6 | 14.07-14.47 | 33 |
| GWAS TRJ | Ge5 | 6 | 14.33-14.73 | 31 |
| GWAS TRJ | Ge4 & Ge5 | 6 | 14.46-14.86 | 28 |
| GWAS TRJ | Ge6 | 6 | 15.71-16.11 | 32 |
| GWAS TRJ | Ge4 | 6 | 16.06-16.46 | 30 |
| GWAS TRJ | Ge6 | 6 | 16.12-16.52 | 29 |
| GWAS TRJ | Ge6 | 7 | 24.92-25.32 | 46 |
| GWAS TRJ | Ge4 | 7 | 27.59-27.99 | 59 |
| GWAS TRJ | Ge6 | 8 | 1.43-1.83 | 62 |
| GWAS TRJ | Ge6 | 8 | 5.84-6.24 | 46 |
| GWAS TRJ | Ge4, Ge5 & Ge6 | 10 | 17.14-17.54 | 45 |
| GWAS TRJ | Ge5 | 10 | 17.21-17.61 | 23 |
| GWAS TRJ | Ge6 | 11 | 15.25-15.65 | 32 |
| GWAS TRJ | Ge5 | 11 | 24.57-24.97 | 44 |
| GWAS TRJ | Ge6 | 11 | 25.38-25.78 | 31 |
| GWAS TRJ | Ge4 & Ge6 | 12 | 25.69-26.09 | 64 |
| GWAS TRJ | Ge4 & Ge5 | 12 | 25.9-26.3 | 49 |
