# Supplementary material for: Biallelic and Genome Wide Association Mapping of Germanium Tolerant Loci in Rice (Oryza sativa L.)
Source: PLoS One. 2015 Sep 10;10(9):e0137577. doi: 10.1371/journal.pone.0137577 (PMC4565582; doi:10.1371/journal.pone.0137577)
Supplement: S5 Table — (DOCX) [file pone.0137577.s009.docx]

Supplementary table 5. Co-localisation of SNPs significantly associated with germanium induced lesions and QTLs for rice arsenic content and silicon content.

| GWA association | | | | | |  |
| --- | --- | --- | --- | --- | --- | --- |
| Trait | Analysis | Chromosome | Mbp | *P* value | SNP ID | Co-localisation |
| Ge6 | ALL | 1 | 23.36 | 4.07E-05 | id1013420 | Husk silicon QTL (Dia *et al.,* 2008) |
| Ge5,6 | ALL | 1 | 27.10 | 7.26E-06 | id1015789 | Husk silicon QTL (Dai *et al.,* 2005) |
| Ge4 | *TRJ* | 1 | 1.4 | 7.07E-05 | id1001153 | Ba×Az germanium lesion QTL (this study) |
| Ge6 | *IND* | 2 | 17.21 | 1.66E-05 | id2006855 | Grain Arsenic QTL (Zhang *et al*., 2014); Ba×Az germanium lesion QTL (this study) |
| Ge6 | ALL | 3 | 32.40 | 3.30E-05 | id3015629 | Ba×Az germanium lesion QTL (this study) |
| Ge4 | *TEJ* | 3 | 12.20 | 3.95E-05 | id3006236 | Grain Arsenic QTL (Zhang *et al*., 2014) |
| Ge4,5 | ALL | 6 | 5.21 | 8.12E-05 | id6003502 | Grain methylated arsenic QTL (Kuramata *et al*., 2013); |
| G4 | *TEJ* | 6 | 8.08 | 4.20E-05 | id6005251 | Grain methylated arsenic QTL (Kuramata *et al*., 2013) |
| Ge6 | *TRJ* | 8 | 1.62 | 3.84E-05 | id8000422 | Grain methylated arsenic QTL, (Kuramata *et al.,* 2013 |
| Ge6 | *TRJ* | 8 | 1.63 | 3.84E-05 | id8000432 | Grain methylated arsenic QTL (Kuramata *et al*., 2013 |
| Ge6 | *TRJ* | 8 | 1.64 | 7.45E-06 | id8000442 | Grain methylated arsenic QTL (Kuramata *et al*., 2013 |
